# ASD-similar social behaviour scores affect stimulus generalization in family dogs

**DOI:** 10.1038/s41598-024-69610-1

**Published:** 2024-08-10

**Authors:** Dorottya J. Ujfalussy, Anna Gergely, Eszter Petró, József Topál

**Affiliations:** 1 Institute of Cognitive Neuroscience and Psychology, HUN-REN Research Centre for Natural Sciences, Magyar Tudósok krt. 2, Budapest, 1117 Hungary; 2 NAP 3.0 Comparative Ethology Research Group, Budapest, Hungary; 3 Department of Ethology, Eötvös Loránd University, Budapest, Hungary

**Keywords:** Generalization, Dog, Social competence, Minor differences, “ASD-like” behaviour, Psychology, Diseases

## Abstract

Generalization, the tendency to respond in the same way to different but similar stimuli, is one of the main cognitive abilities that make category formation possible and thus is a prerequisite for efficiency in learning. Individuals with autism spectrum disorder (ASD) experience pervasive difficulty with producing generalized responses across materials, people, places, and contexts. Increasing evidence suggests that “ASD-like” social impairments appear endogenously and spontaneously in family dogs providing a high-validity model for understanding the phenotypic expression of human ASD. The present study aims to further investigate the dog model of ASD by the approach of searching for analogues in dogs showing “ASD-like” social impairments of cognitive phenomena in humans specific to ASD, specifically impairments of generalization abilities. We have tested 18 family dogs with formerly established “ASD-like” behaviour scores (F1, F2, F3) in a generalization task involving three conditions (size, colour and texture). We found a significant association between F1 scores and test performance as well as improvement during testing sessions. Our study provides further support for the notion that dogs with lower social competence—similarly to humans with ASD—exhibit attentional and perceptual abnormalities, such as being sensitive to minor changes to a non-adaptive extent.

## Introduction

In psychology, stimulus generalization is defined as the tendency for a conditioned response to be elicited by stimuli that are not identical, but similar to the original conditioned stimulus^1^. The so-called generalized response, elicited by a similar stimulus is usually slightly less distinct than the one elicited by the original conditioned stimulus and will diminish once the new stimulus departs increasingly from the original^2^. Generalization is made possible by the ability to disregard negligible differences, and it is a prerequisite of some more advanced cognitive skills, such as category formation and observational learning^3^. Our ability to engage in stimulus and response generalization spares us the practically impossible task of direct training in each and every individual skill, thus difficulties in producing generalized behaviour compromise cognitive function^4^. For example, individuals with autism experience extensive difficulty with producing generalized responses (i.e. disregarding minor, negligible changes irrelevant to situation) across materials, people, places, and contexts^5–7^, seriously impairing their ability to operate independently.

Autism Spectrum Disorder (ASD) is a neurodevelopmental disorder that influences communicative and social abilities, affecting individuals in a variety of ways, with varying degrees of severity^8^. People with ASD might experience sensory sensitivities and/or engage in repetitive movements. The causes and mechanisms of this neurodevelopmental disorder are largely unknown, so both treatment and early intervention are challenging. Individuals with autism exhibit attentional and perceptual abnormalities, such as noticing minor changes to a non-adaptive extent^9^. This acute ability to process fine detail^10–14^ and related difficulty in recognizing generic similarity among objects or contexts is suggested to underlie generalization and category formation deficits.

Various theories have been put forward to explain the underlying mechanisms of ASD which lead to a wide range of complex symptoms^15–17^. Some of the most recent theories are in line with the above-described findings on generalization. The so-called “aberrant precision account of ASD” suggests that atypical sensory processing inherent to ASD may impair the ability to disregard minor differences, causing impaired performance in placing two objects into the same category based on similarities and disregarding irrelevant variability (as “noise”) (for review see:^18^).

Recently, the usefulness of a family dog model in autism research has been suggested^19–21^. This model has been proposed to have higher translational validity than traditional rodent models, as dogs’ social-cognitive abilities have evolved in the human niche and in many aspects have been shown to be similar to those of human children^22–24^. Moreover, unlike in rodent models, where symptoms often need to be experimentally induced (e.g.^25^, “ASD-like” social impairments such as owner-directed explosive aggression, trancing, and social withdrawal, seem to appear endogenously and spontaneously in family dogs^20,21,26,27^ providing higher validity in understanding phenotypic expression, as well as possible underlying mechanisms.

However, the suggested family dog model of ASD remains to be tested, although some empirical evidence for its validity has already been found, with results corresponding to those found earlier in humans with ASD (see e.g. differential attention to social vs. non-social stimuli;^28^). The present study aims to further test the dog model of ASD by searching for analogues in dogs that exhibit “ASD-like” social impairments and cognitive phenomena specific to ASD in humans, specifically impairments of generalization abilities.

Stimulus generalization has been studied extensively in dogs, from the early works of Pavlov^29,30^ to the most recent studies of olfactory generalization in scent detection dogs (e.g.^31–33^. However, the generalization of trained target objects and their properties seems surprisingly understudied. Van der Zee and colleagues^34^ investigated the generalization of a word referring to an object to other objects of the same shape, size or texture. Their single subject was a special dog, Gable, who has been shown to reliably know the word for 43 different objects. In humans, stimulus generalization shows a characteristic shape bias, meaning that both toddlers and adults give more weight to shape than size or texture when generalizing or categorizing^35^. The word generalization study of van der Zee and team^34^ aimed to verify whether this specific dog showed a human-like shape bias in generalization, and they found that it did not. Importantly, however, Gable generalized to size, and after a longer exposure also to texture.

Based on the classical and more recent evidence above, it is reasonable to assume that dogs in general are capable of stimulus generalization. The question remains, however: is this ability impaired in dogs showing “ASD-like” behavioural elements similar to humans with ASD? To explore this, we used the recently developed Interspecific Social Responsiveness Survey (ISRS—^28^) to assess factor scores corresponding to social skills that may be linked to ASD-like behaviours in a group of pet dogs. The ISRS was developed with the aim of evaluating the presence and intensity of social behavioural issues similar to those observed in human autism spectrum disorder in dogs. This assessment tool was constructed based on the diagnostic criteria utilized for human ASD. In addition to questions concerning non-social symptoms, the ISRS incorporates questions from six distinct categories, corresponding to social behavioural symptoms outlined in the Diagnostic and Statistical Manual of Mental Disorders, DSM-V (skills related to communication with and attention to humans, emotional and behavioural synchrony, sociability and attachment to humans). Each of these six social categories is represented by 4–5 questions in the ISRS, which can be assessed using a 5-point Likert scale. The social behavioural segment of ISRS can be categorized into three factors each representing different aspects of dogs’ social competence (*Contact seeking and synchronization-F1, 10 items; Behaviour toward strangers-F2, 4 items; Attention to human communicative signals- F3, 6 items*—see^28^ for more details). Then we tested these dogs in a generalization task and analysed the potential impact of their scores on task performance. We predicted that dogs with poor social competence (i.e., individuals with ‘ASD-like traits) would experience more difficulties in generalizing the learned response in an object-label association task. More specifically, we hypothesized that dogs characterized by (i) weaker susceptibility to emotional/behavioural contagion, (ii) a lower tendency to seek social contact with humans and (iii) less willingness to initiate social interaction with people would show systematic differences from highly competent dogs in terms of how they generalize their response to situations where “one of the characteristic features” (size, colour or texture) of the target object is changed.

## Methods

### Ethics statement

This research was approved by the National Animal Experimentation Ethics Committee (Ref. No. PE/EA/55-4/2019). The research was done following the Hungarian regulations on animal experimentation and the Guidelines for the use of animals in research described by the Association for the Study of Animal Behaviour (ASAB). Dogs and their owners were recruited on a voluntary basis, and all owners gave informed consent.

### Subjects

**Figure 1:**
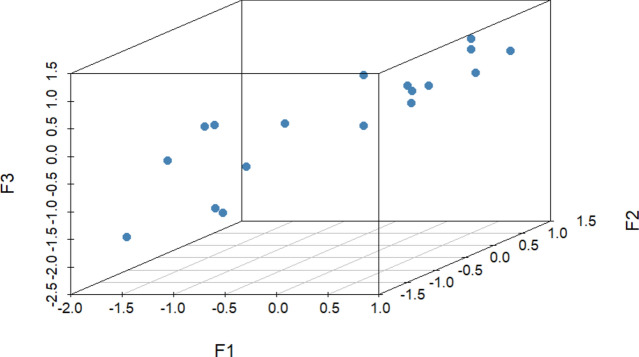
3-D representation of the distribution of subjects based on the 3 ISRS factor scores (F1: Contact seeking and synchronization; F2: Behaviour toward strangers; F3: Attention to human communicative signals).

18 healthy adult pet dogs (mean age ± SD: 6.1 ± 2.8 years; 10 males and 8 females from various breeds, see Subject Table S1 in Supplementary materials for details) with a relatively wide range of ISRS scores (Fig. 1) and their owners participated in the study. It is important to note that all 18 dogs fulfilled the learning criterion and were included in the analysis (see Procedure for details).

### Experimental arrangement

**Figure 2:**
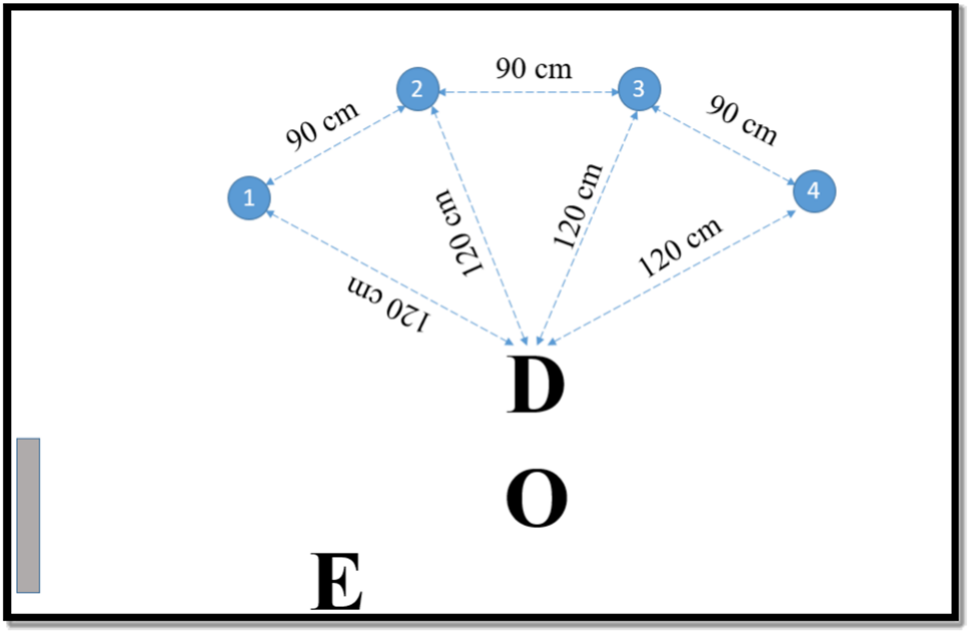
Experimental arrangement. Blue circles represent the objects’ location (1–4) for the Training and Test phases. E = position of the Experimenter, O = position of the owner, D = starting position of the dog. The grey rectangle shows the location of the door used in the experiment.

Dogs were tested in a laboratory room of the Research Centre for Natural Sciences (5 m × 6 m) with tape on the floor marking standardized locations of the experiment (Fig. 2). A video camera was mounted on each wall, with output recorded on a computer.

In order to examine stimulus generalization, we used different objects in the present experiment. One of them was used as the assigned target object and we trained the dogs to be able to choose this object instead of the other non-target objects during the training phase. Then, during the test phase, we changed different properties of these objects (i.e. colour, size or texture) and we tested if dogs were still able to choose the target-like object.

The basic set of objects comprised the following six items: a purple carton box (length 16 cm × width 16 cm × height 11 cm), a red wooden skittle (7 × 7 × 16), a grey metal cup (11 × 9 × 8), a yellow plastic spoon (16 × 4 × 3), a white leather shoe (13 × 6 × 5) and a beige plush pencil case (21 × 7 × 3).

**Figure 3:**
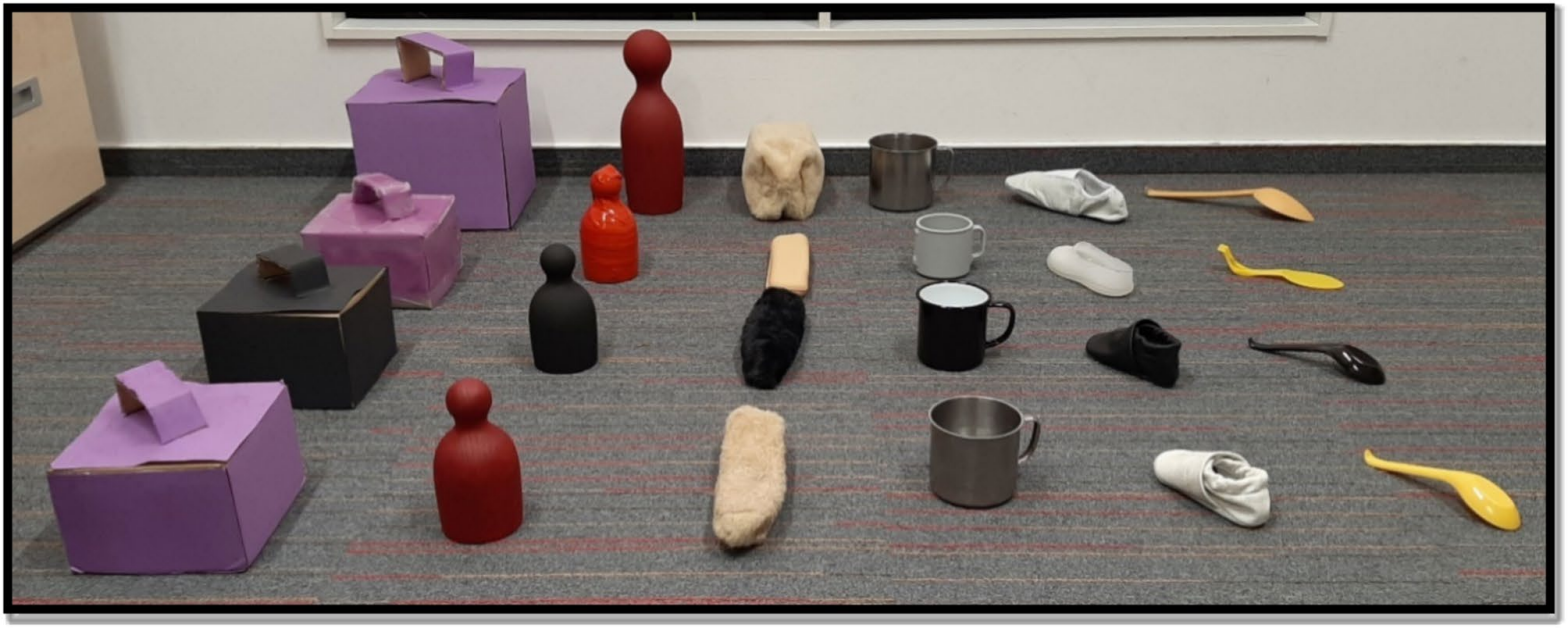
Objects of the experiment. First row: Basic set of objects used in the Preference, Pre-training, Training and Test warm-up trials. Second row: objects used in the ‘Colour change’ condition. Third row: objects used in the ‘Texture change’ condition. Fourth row: Objects used in the ‘Size change’ condition. Objects’ category names from the left to the right: box, skittle, case, cup, shoe, spoon.

In the ‘Colour change’ condition the objects were identical to those in the basic set, except that all of them were black. In the ‘Texture change’ condition the objects were identical to those in the basic set, except that all of them were made of silicon and in the ‘Size change’ condition the colour and texture of the objects remained unchanged compared to the basic set of items, but all of them were larger (with a width x height x depth at least 2 times greater—see Fig. 3).

### Procedure

**Figure 4:**
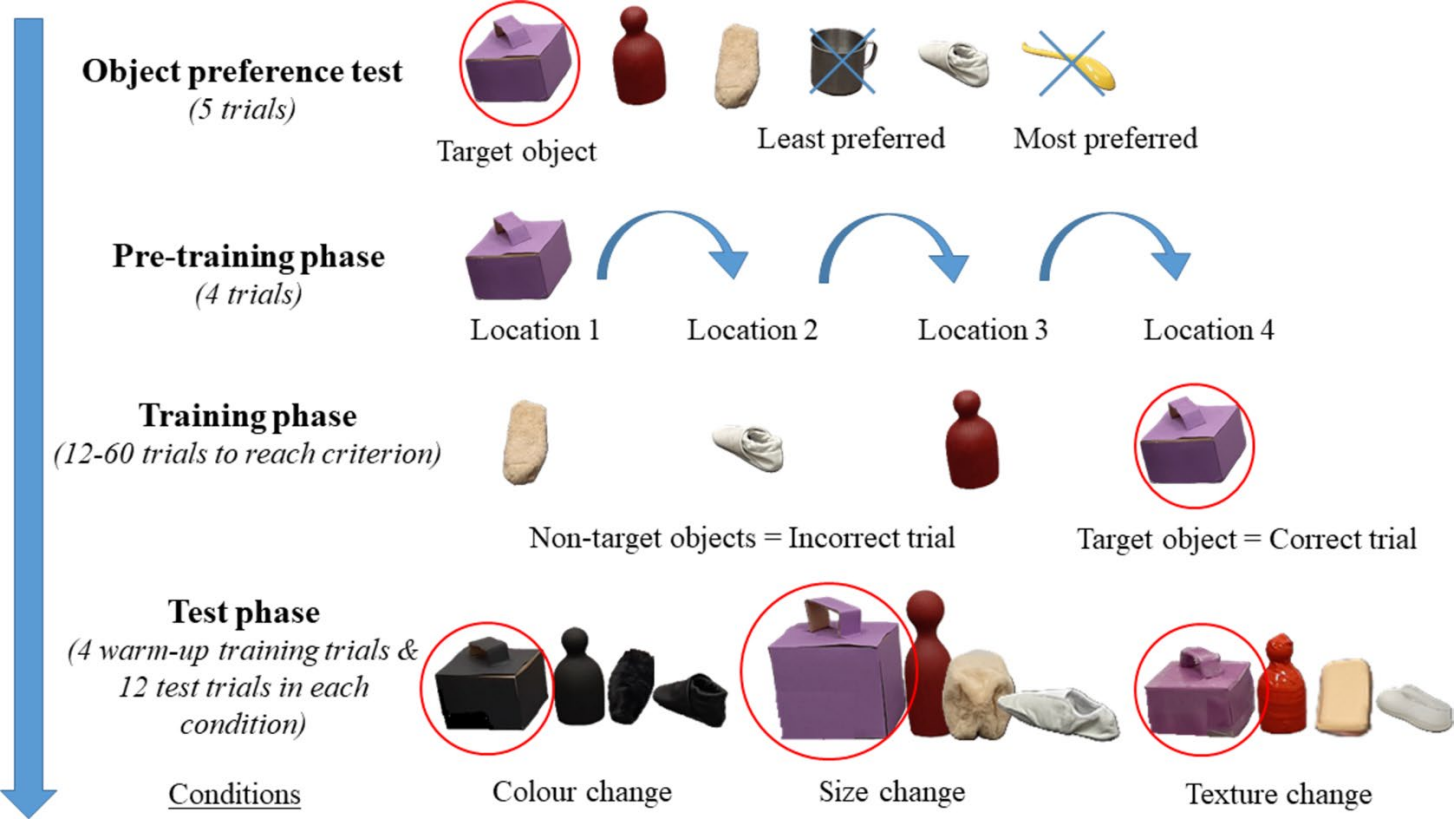
Summary of the procedure with example target- and non-target objects.

The study consists of four phases: the Object-preference test, the Pre-training phase, the Training phase, and the Testing phase (Fig. 4).

#### Object-preference test

This phase aimed to investigate dogs’ spontaneous preference/avoidance toward the six pre-selected ‘basic objects’ (see Fig. 3) and consisted of a maximum of 5 trials. During the first trial, dogs had an opportunity to explore all six objects placed on the floor in a half-circle and random order while the owners were encouraging them to approach and/or retrieve any object and praise them if they did so. When the dog approached, explored and/or manipulated one object longer than 3 s the Experimenter (henceforth E) asked the owner to praise the dog and call him/her back to the starting point. Then E removed this particular object while leaving the 5 remaining objects on the floor. Then the dog was allowed (and encouraged) to approach and explore the objects again. We continued this process until only one object was left on the floor. The most-preferred (i.e. first removed) and the least-preferred (i.e. not removed) objects were excluded from the subsequent experimental phases. The remaining four objects were included in the training. Note that one of these objects was randomly designated as a “target object” and the other three were designated as “non-target” objects.

#### Pre-training phase

This phase aimed to teach dogs to (i) indicate the target object (ii) and that the target object can be placed in different places in the room. In this phase, E placed the target object in front of the dog to one of the four predetermined object-location points (see Fig. 1). After placing the target object, E stood back to her place and turned her back to the dog to avoid any eye contact with him/her. Then the owner gave the Command (see Table 1) and released the dog. If the dog indicated the target object, it was Praised (see Table 1). Then E placed the target object to another object-location point and the procedure was repeated until the dog successfully indicated the target object at least once in all four object-location points. If the dog did not indicate the target object in 20 s. or if the dog only approached but did not indicate the target object, the owner called his/her dog back to the start point. Then E applied the Attention-getting procedure (see Table 1). Then, the owner issued the command once more and released the dog.

**Table 1:** List of phases and their definition used in the experiment.

Phase	Definition
Indication	The dog approached an object within 20 cm and remained there while orienting towards it for 2–3 s. OR The dog touched an object with its nose/muzzle/paw or mouthed it (see Table S1 for details)
Correct trial	The dog indicated the target object
Incorrect trial	The dog indicated a non-target object
Praise	After correct trials, the dogs were praised by the experimenter (E) and the owner verbally, while E walked directly to the target object and gave a piece of a treat to the dog
Attention-getting procedure	E approaches the target object and lifts it while she is using ostensive communicative signals (saying: ‘*Look how cool is this!*’ ‘This is ‘{*category name of the target object*}!’ ‘*This is super!*’) while keeping eye contact with the dog and using a high-pitched voice
Giving a command to the dog by using ‘category names’	*‘Search for the ‘{category name of the target object*}!’ Category names: ‘*box*’, ‘*skittle*’, ‘*case*’, ‘*cup*’, ‘*shoe*’, ‘*spoon*’

At this point, it is important to note that we did not train a specific indication behaviour to the dogs. Our only criterion was that the indication behaviour had to be performed in close proximity (i.e. within 20 cm) to the chosen object. Besides this criteria, all dogs were allowed to develop an individual but consistent indication behaviour during the pre-training and training phases (see Table 1 and Table S1 for details).

#### Training phase

In this phase, dogs were trained to select the named target object when three non-target objects were also present. Training sessions occurred once or twice a week (with a minimum of 3 and a maximum of 10 days between two consecutive sessions) by E until the dog reached the learning criterion. The learning criterion was set as at least 10 correct responses out of 12 trials. Dogs achieved this predetermined criterion after completing 12–60 training trials (mean ± SD, 32 ± 13 trials, see Table S1 for details). Dogs participated in 1–3 training sessions on each occasion and each session consisted of 12 training trials (Table S1).

In one training trial, dogs were presented with the target object and three non-target objects (arranged by E on the floor in a half-circle in a counterbalanced order). After placing the objects, E stood back to her predetermined place and turned her back to the dog to avoid any eye contact. Then the owner commanded the dog (named the target object) without pointing or looking directly at the objects. If the dog selected the target object, it was praised. However, if the dog selected a non-target object, the owner commanded the dog again (naming the target object) without calling it back. If the dog now indicated the target object, it was praised, but if stayed next to the non-target object or indicated another non-target object the owner kindly called the dog back and the trial was repeated without replacing the objects.

After two incorrect trials, E applied the ‘Attention-getting’ procedure (see Table 1), and then the owner commanded the dog to approach the target object again and let it free. If the trial was still incorrect (this happened only once during the experiment), the owner kindly called the dog back while E removed all non-target objects from the half circle. Then the trial was repeated with the target object only (as in the Pre-training phase).

Between trials E changed the positions of the objects in the half-circle using the following algorithm: she changed the position of the target object so that it alternately switched places with the neighbouring non-target object on the left or with the second non-target object on the left. E was also careful about the last object she touched (i.e. she randomized which objects were touched last). The starting position of the target object in each training session was counterbalanced between and within dogs.

#### Test phase

After reaching the learning criterion, the dog was moved to the test phase. We applied three test conditions, each took place on different days (minimum 3, maximum 10 days in between) and started with 4 warm-up trials that were completely identical to the training trials (i.e. to refresh dogs’ memory on the task before the test trials). Dogs then were again instructed to search for the target object from an array of 4, but this time one of the characteristic features of the objects was changed (‘colour change’, ‘size change’ and ‘texture change’ conditions—see above).

E then changed the positions of the objects in the half-circle and the owner commanded the dog to search for the target object again. The position of the target object was changed from trial to trial using the same algorithm as was used in the *Training phase.* E was also careful about the last object she touched (i.e. she randomized which objects were touched last. The starting position of the target object in each training session was counterbalanced between and within dogs. After placing the objects, E stood back in her place and turned her back to the dog to avoid eye contact or any other unconscious cueing.

Dogs participated in the three conditions (size-, colour-, and texture change) on different days with a 3–10-day break in between (for details see Table S1). Each condition included 4 warm-up trials and 12 test trials. The order of size-, colour-, and texture change conditions was randomized between subjects (Fig. 4, Table S1).

Note that test trials were conducted similarly to the training trials in terms of object placement, object position changes, commands and praising. The only difference between the training and test trials was that the owner kindly called the dog back to the starting point after an incorrect trial without allowing the dog to self-correct.

### Data analysis

All subjects’ factor scores we calculated based on owner reports using the Interspecific Social Responsiveness Survey (ISRS—^28^). Responses to this questionnaire break down into three factors representing different aspects of dogs’ social competence: F1—Contact seeking and synchronization, F2—Behaviour toward strangers, and F3—Attention to human communicative signals.

Dogs’ choice behaviour (correct/incorrect; 1/0) was recorded and analysed using Solomon Coder software (András Péter, http://solomoncoder.com). Inter-observer agreements for subjects’ choice behaviour were assessed by means of parallel coding of 15% of the total trials by two observers. The inter-observer reliability was excellent (Cohen’s kappa = 0.96).

The association between ISRS factors scores (F1, F2, F3) and learning acquisition during training was analysed with ordinal regression (the total number of training trials necessary to reach criterion as dependent variable and F1-3 scores as predictor covariates). The number of correct trials during training was also analysed with Binomial GLMM (SPSS version 25). The model included the ISRS factor scores (F1, F2, F3) and their two-, and three-way interactions.

In order to examine, whether ISRS factors scores (F1, F2, F3) were associated with pre-test performance, we also analysed the four warm-up training trials conducted before each test condition (see Methods, Procedure, *Test phase* for details). We applied ordinal regression, wherein the number of correct pre-test trials for each dog before every test condition was included as the dependent variable with F1-3 scores serving as predictor covariates.

Analysing the test trials, we first conducted one-sample Wilcoxon signed rank tests to determine if dogs’ performance in the initial four test trials (1–4) of various test conditions (i.e. *Colour change, Texture change, Size change*) significantly differed from the performance established as the learning criterion (83.3%). Then we used Binomial GLMM analyses to check for any effect of ISRS factor scores (F1, F2, F3), condition (size, colour or texture changes), as well as their two-way interactions on dogs’ performance. We then similarly analysed the first four (1–4) and last four (9–12) test trials separately with Binomial GLMMs to search for the initial effects of ISRS factor scores on dogs’ generalization ability and possible effects of training during the testing sessions. Finally, we calculated the change in dogs’ performance (delta) between the first four (1–4) and the last four (9–12) test trials and used ordinal regression analyses (the improvement in performance during testing—delta scores—between the first and the last four trials as dependent variable and F1-3 scores as predictor covariates), to determine if improvement during testing was associated with ISRS factor scores.

In all GLMM models, the subjects’ ID (identity number) as a random factor was included to control for repeated measurements. Non-significant effects were removed from the model in a stepwise manner (backward elimination technique). For post hoc tests, Bonferroni corrections were used. Statistical tests were two-tailed, α value was set at 0.05.

## Results

### Association between ISRS factors scores and learning acquisition during training and performance in warm-up trials

There were no significant associations between F1, F2 and F3 scores and the total number of trials necessary to reach the learning criterion (parameter estimate(F1): -0.842, SE: 0.477, χ^2^_(1)_ = 3.283, *p* = 0.078; parameter estimate(F2): 0.102, SE: 0.556, χ^2^_(1)_ = 0.031, *p* = 0.861; parameter estimate(F3): − 0.534, SE: 0.463, χ^2^_(1)_ = 1.513, *p* = 0.219). GLMM analysis showed no significant three- and two-way interactions of the ISRS factors, therefore interactions were excluded from the model. During backward modelling, F2, and then F3 scores were eliminated from the model because of their non-significant effects. The final model included only the F1 score, but its effect was also non-significant (GLMM, F_1,572_ = 1.604; *p* = 0.206). This means that ISRS factors did not affect dogs’ performance during training trials.

Pre-test performance (as measured by the number of correct choices during the 4 warm-up training trials before test trials, see Methods, Procedure, *Test phase*) showed no significant association with any of the three ISRS factor scores—all subjects performed similarly well (ordinal regression, parameter estimate(F1): 0.251, SE: 0.467, χ^2^_(1)_ = 0.295, *p* = 0.587; parameter estimate(F2): − 0.297, SE: 0.597, χ^2^_(1)_ = 0.24, *p* = 0.624; parameter estimate(F3): − 0.087, SE: 0.478, χ^2^_(1)_ = 0.036, *p* = 0.849).

### Dogs’ performance in terms of correct choices in the different test conditions

As we cannot entirely disregard the possibility of learning through experience during the testing sessions, we considered the first four (1–4) trials as most indicative of spontaneous generalization ability. We found that dogs’ performance in the initial four test trials (1–4) showed no significant difference from the performance established as the learning criterion (83.3%), indicating that they were able to generalize the learned response to objects with different colours, sizes or textures. More specifically, the average success rate of dogs in the *Texture change* condition was 73.6% (one sample Wilcoxon signed rank test, median = 3, IQT = 0.5, *p* = 0.114), in the *Colour change* condition the average success rate was 75% (median = 3, IQT = 0.25 *p* = 0.158), while in the *Size change* condition a 83.3% average success rate was found (median = 3, IQT = 1.0, *p* = 0.841). Note, that dogs also exhibited a similar performance during the last four test trials (test 9–12) to the warm-up phase in each condition (*Texture change*: median = 3, IQT = 0.5, p = 0.210; *Size change*: median = 3, IQT = 1.0, *p* = 0.842; *Colour change*: median = 3, IQT = 0.25, *p* = 0.842).

**Figure 5:**
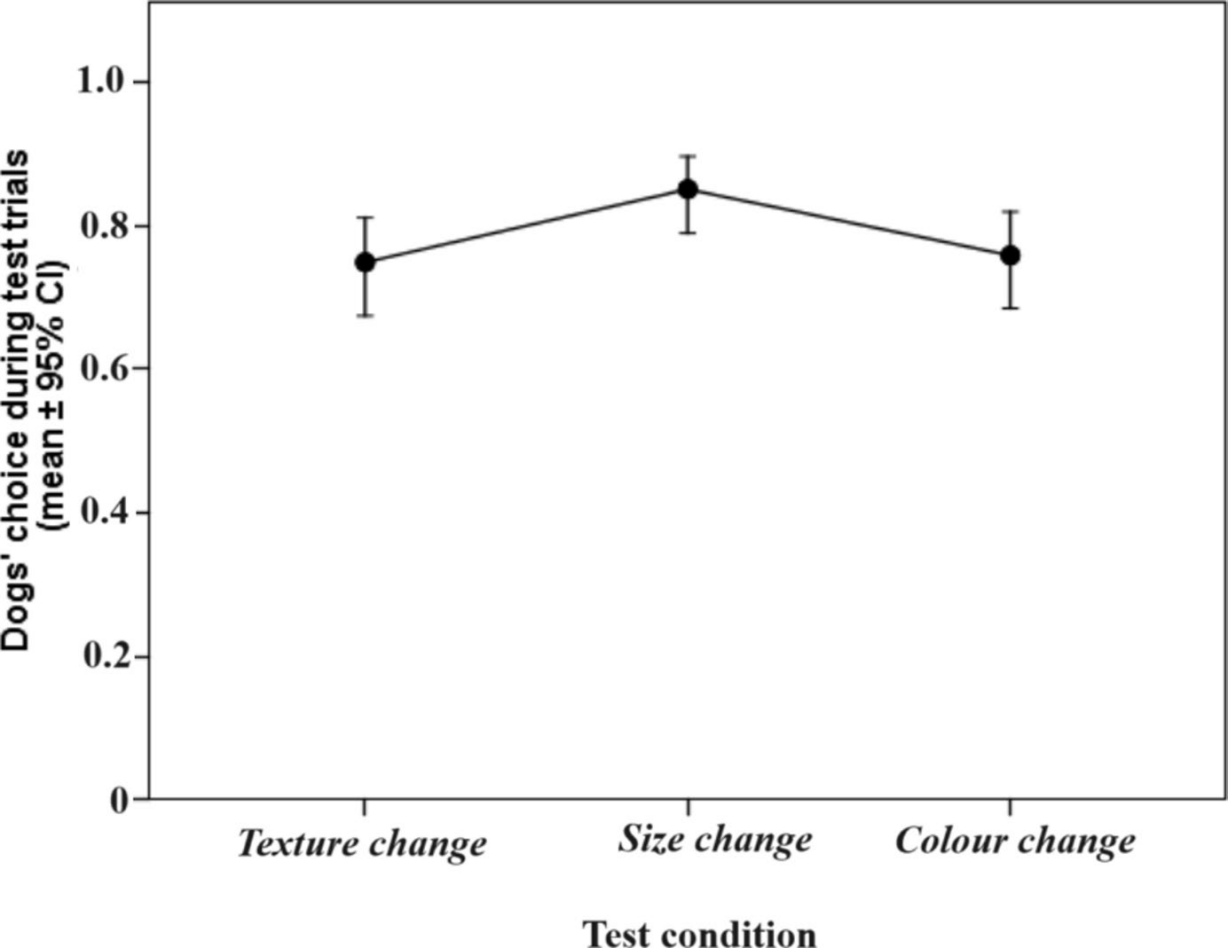
Dogs’ performance during test conditions.

**Figure 6:**
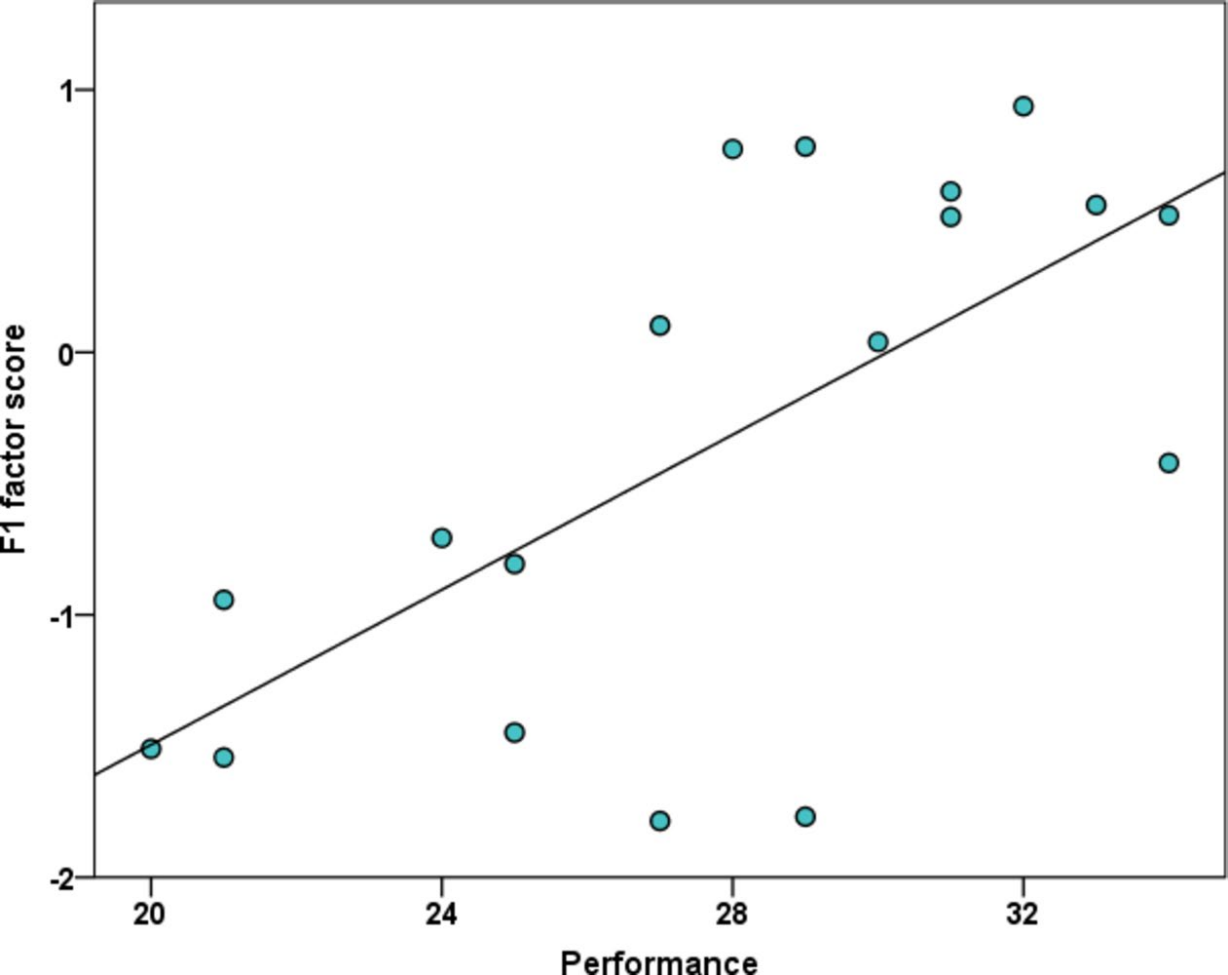
Association between the total number of correct choices during the test trials in the three different conditions and F1 factor scores extracted from ISRS questionnaire (GLMM, F_1,644_ = 11.898; *p* = 0.001).

From the Binomial GLMM on subjects’ performance during test trials, the non-significant interaction and main effects were eliminated in the following order: F2 × F3, F1 × F3, F3 × condition, F1 × condition, F1 × F2, F2 × condition, F2, F3. The final model showed that the test condition had a significant effect on dogs’ performance (GLMM, F_2,644_ = 4.031; p = 0.018). Post-hoc tests revealed better performance in the *Size change* condition than in the *Texture-* (*p* = 0.026; *SE* = *0.039; t* = 2.631) and *Colour change* (*p* = 0.032; *SE* = 0.038; *t* = − 2.412) conditions, while no difference was found between the latter two conditions (*p* = 0.822; *SE* = 0.042 *t* = 0.226, see Fig. 5). The final GLMM also showed a significant effect of F1 scores (*Contact seeking and synchronization)* on dogs’ overall performance during the 3 × 12 test trials (GLMM, F_1,644_ = 11.898; *p* = 0.001); higher F1 scores were associated with higher performance (Fig. 6).

When analysing the first four (1–4) test trials separately, we did not find any significant interaction or main effects of any of ISRS factor scores, and the effect of condition is also undetectable here (the final model included the F1 score as main effect, F_1,214_ = 3.771, *p* = 0.053). When analysing the last four (9–12) test trials separately, we eliminated the non-significant interactions and main effects in the following order: F3 × condition, F1 × condition, F2 × condition, F2 × F3, F1 × F3, F1 × F2, F2, F3, condition. The final model revealed significant effect of the F1 score (*Contact seeking and synchronization,* F_1,214_ = 14.661, *p* < 0.0001); higher F1 scores were associated with better performance.

**Figure 7:**
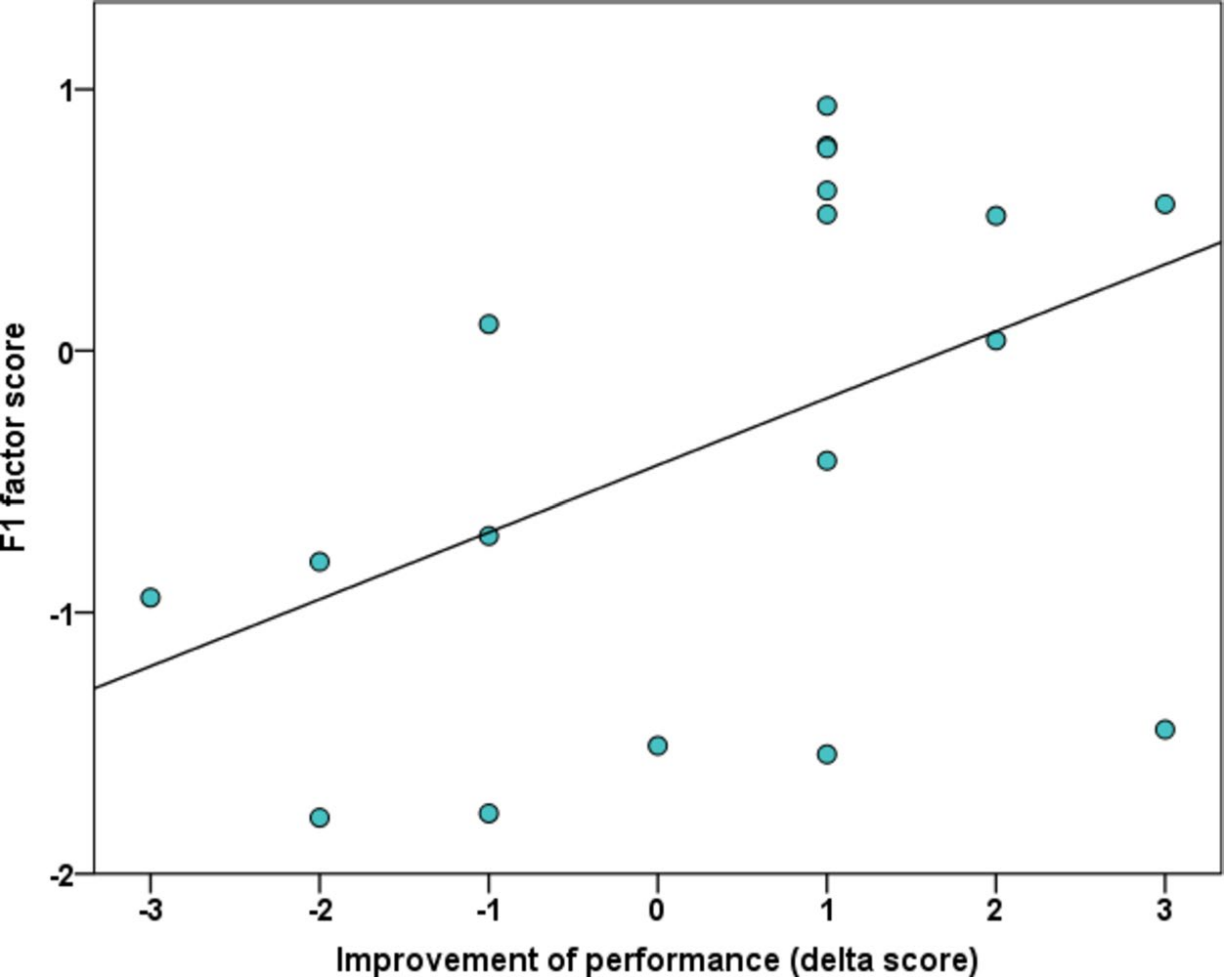
Association between delta scores during test trials (i.e. difference in performance between the last- and first four test trials) and ‘Contact seeking and synchronization’ (F1) scores extracted from ISRS questionnaire (parameter estimate(F1): 0.914, χ^2^_(1)_ = 3.9, *p* = 0.048).

Finally, we found that the improvement of performance during testing, as measured by the difference in correct trials (delta scores) between the first- and the last four trials was significantly positively associated with the F1 score (parameter estimate(F1): 0.914, SE: 0.491, χ^2^_(1)_ = 3.9, *p* = 0.048—see Fig. 7), while the link between delta and F3 scores (parameter estimate(F3): 0.785, SE: 0.482, χ^2^_(1)_ = 3.252, *p* = 0.071), and delta and F2 score (parameter estimate: 0.763, SE: 0.589, χ^2^_(1)_ = 1.748, *p* = 0.186) were non-significant.

## Discussion

In order to address our main question—whether, similarly to humans with ASD^5–7^, generalization ability is impaired in dogs with higher scores on “ASD-like” behaviours—we first had to rule out the possibility that low scoring (i.e. socially low functioning) dogs are, in some way, hindered in learning the target object and reaching training criterion. The finding that there was no association between ISRS factor scores and learning performance during training and warm-up trials—meaning the ability to learn to select a target object from an array in a social training context is not affected by any of the factor scores relating to “ASD-like” behaviour—rules out this possibility.

Moreover, we managed to find further evidence that dogs in general are indeed able to disregard minor differences and spontaneously generalize the learned response to similar, but not identical objects in all conditions, as we found that performance in the first four (1–4) trials of generalization tests (i.e. spontaneous performance, as described above in Results) did not differ significantly from criterion performance on the group level. While this was true in all conditions, we found that dogs performed significantly better in the “size change” condition than in the “texture change” and “colour change” conditions. The finding, that dogs generalize easier to size than other object properties (such as texture) is also in agreement with similar findings of van der Zee and colleagues^34^, who reported their single subject to generalize to size most readily. The slight, but not significant setback from criterion performance to performance in the first four (1–4) trials, which could be detected in the Texture and Colour conditions are indicative of the recognition of the implemented negligible differences.

When checking for an association of ISRS factor scores with performance over all test trials, we found a positive relationship between the F1 score (*Contact seeking a synchronization*) and subjects’ performance in generalization test trials, with higher scoring individuals showing better performance. Thus, low F1 scores seem to be associated with poorer generalization abilities. We analysed the first and last four trials separately in an attempt to assess the effects of “ASD-like” behaviour traits not only on spontaneous generalization ability (trials 1–4), but also on improvement during the testing sessions (trials 9–12). The positive relationship was not found when analysing the first four trials (1–4) separately but was also detectable when analysing the last four (9–12) trials separately. Interestingly, we also found that the improvement of performance over the test phase (as measured by our delta scores) was also significantly positively correlated with the F1 score, suggesting that low scoring individuals are also less able to improve their performance over the test trials.

While we cannot entirely rule out the possibility that improvement during testing (as measured by the difference between first four trial performance and last four trial performance) may be mediated by the subjects’ social competence and ability to (re)learn during testing trials in a social context, we find this improbable, given that our subject were all family dogs accustomed to indoor social/training settings and even more importantly, the lack of associations between ISRS factor scores and learning performance during training and warm-up trials. As training, warm-up and testing trials were very similar, rather we suggest that while dogs performance drops in the first four test trials possibly indicating that they notice the implemented changes, high F1 scoring individuals gradually improve in disregarding the minor changes, while low F1 scoring individuals may not be able to do so Thus we have reason to think that the reported effect is due to a lower tendency in low scoring individuals to disregard negligible differences, rather than being less able to function in a social context.

A further limitation of this study is that dogs’ social competence is assessed indirectly through an owner questionnaire, thus results are subject to owners’ judgment. A possible further step would be to add experiments directly testing social competence prior to the generalization study. It also must be noted that subject numbers are not very high. Testing more dogs in this paradigm could potentially strengthen our results.

In summary, our results are in line with earlier findings^29–33^ showing evidence that dogs are capable of stimulus generalization, which can also be detected in the relatively understudied object property domain^34^.

Our study provides further support for the notion that dogs with lower social competence—similarly to humans with ASD—exhibit attentional and perceptual abnormalities, such as noticing minor changes to a non-adaptive extent. This acute ability to process fine detail and related difficulty in recognizing generic similarity among objects or contexts makes generalization and thus categorization difficult. As “ASD-like” social impairments appear endogenously and spontaneously (meaning they appear without being artificially induced) in family dogs, we suggest that they provide a high validity model in understanding phenotypic expression, as well as possible underlying mechanisms of human ASD.

### Ethical note

We confirm that our study is reported in accordance with ARRIVE guidelines on the full and transparent reporting of research involving animals.

### Supplementary Information

Supplementary Tables.

## Data Availability

All data generated or analysed during this study are included in this published article [and its supplementary information files].
